# Polystyrene negative resist for high-resolution electron beam lithography

**DOI:** 10.1186/1556-276X-6-446

**Published:** 2011-07-12

**Authors:** Siqi Ma, Celal Con, Mustafa Yavuz, Bo Cui

**Affiliations:** 1 Waterloo Institute for Nanotechnology (WIN), University of Waterloo, 200 University Ave. West, Waterloo, ON N2L 3G1, Canada

## Abstract

We studied the exposure behavior of low molecular weight polystyrene as a negative tone electron beam lithography (EBL) resist, with the goal of finding the ultimate achievable resolution. It demonstrated fairly well-defined patterning of a 20-nm period line array and a 15-nm period dot array, which are the densest patterns ever achieved using organic EBL resists. Such dense patterns can be achieved both at 20 and 5 keV beam energies using different developers. In addition to its ultra-high resolution capability, polystyrene is a simple and low-cost resist with easy process control and practically unlimited shelf life. It is also considerably more resistant to dry etching than PMMA. With a low sensitivity, it would find applications where negative resist is desired and throughput is not a major concern.

## 1. Introduction

Electron beam lithography (EBL) [1], focused ion beam (FIB) lithography [2], and nanoimprint lithography (NIL) [3] are currently the three most widely employed nanolithography techniques. Among them, EBL is undoubtedly the most popular for R&D. Unlike NIL, EBL can generate arbitrary patterns without the need of fabricating a mold first. Though not as versatile as FIB, which can do both lithography using a resist and milling, EBL is capable of exposing thick (> > 100 nm) resist without ion contamination to the resist. In addition, it is faster than FIB exposure since the electron beam can remain well-focused below 10-nm beam size even with nA beam current, as is needed for fast writing. In recent years, one main trend in EBL development is the effort being made toward ultra-high resolution and pattern density, with the record pattern density of 9-nm period line arrays [4]. Desirable properties for EBL resist include high sensitivity, high contrast, and high dry etching selectivity to the substrate materials. Positive resist is typically used for EBL, largely because of the availability of the benchmark resist poly(methyl methacrylate) (PMMA) that offers high resolution with low cost and ease of process. With its higher sensitivity and etching resistance than PMMA, ZEP520 (positive-tone, Zeon Corp.) is arguably the second most popular EBL resist.

However, for some applications, such as the fabrication of hole arrays in a metal film (the structure for extraordinary optical transmission [5]) by using liftoff, negative resist would offer substantially shorter exposure time, except when using a more complicated "resist tone reversal" process [6]. Unfortunately, there is no negative resist that gains similar popularity as PMMA and ZEP520. Bilenberg et al. have selected four negative EBL resists and compared their performance: calixarene (Tokuyama Corp.), ma-N 2401 (Microresist Technology), SU-8 (Microchem Corp.), and mr-L 6000 (Microresist Technology) [7]. As chemically amplified resists, SU-8 and mr-L 6000 offer superior sensitivity, but with low contrast and resolution (more strictly speaking, half-pitch for dense periodic line array patterns), which is limited by the diffusion of the photoacid generator during postbaking. Ma-N 2401 has sensitivity comparable to that of ZEP520 resist, but with far inferior resolution. Among the four resists, calixarene offers the highest resolution. Calixarene has been studied as a candidate resist for fabricating using EBL bit-patterned recording media that have achieved areal density of 1.4 and 1.6 Tbits/in^2 ^(corresponding to a dot array of 20-nm period) [8,9] using very thin (sub-20 nm) film. However, it has low sensitivity despite being a chemically amplified resist, and the acid generated in the exposed area may diffuse into the unexposed area, blurring the latent image.

In recent years, hydrogen silsesqioaxene (HSQ) probably attracted more attention than any other negative tone resist [10-12]. HSQ is an excellent inorganic EBL resist that has demonstrated the highest resolution of 9-nm period line array patterns [4,13], thanks to its small molecular size and lack of swelling during development [14]. (Metal halides have actually demonstrated better resolution, but they are not practical resists due to their extremely low sensitivity and inability to form arbitrary patterns [1].) However, in addition to its low sensitivity, HSQ is not suitable for liftoff unless when used with a double layer resist stack, such as HSQ coated on PMMA. The development process is also self-limiting due to crosslinking of resist by the developer, leading to incomplete removal of unexposed resist, though a salty developer can minimize this effect [4,15]. Moreover, HSQ is unstable, and so spin coating, baking, exposure, and development must be done quickly (yet, this is not possible if the exposure time is long) [16].

In addition, all the above resists are commercially formulated with typically high cost and short shelf life. Therefore, it is preferable to have a negative resist like PMMA, which is a simple polymer with low cost and practically unlimited shelf life, and can be dissolved easily using various solvents to give the preferred film thickness. Polystyrene is such a resist, as it undergoes crosslinking when exposed to deep UV light or an electron beam. Previously, dense periodic patterns with 40-nm period lines have been demonstrated using low molecular weight polystyrene resist [17]. In this article, we investigate the ultimate resolution (half-pitch for dense periodic structure) that can be achieved with polystyrene, and demonstrate the patterning of 20-nm-period lines and 15-nm-period 2D dot arrays, which are the highest densities achieved using organic EBL resists (inorganic resists like HSQ and metal halides have achieved higher resolution). Besides ultrahigh resolution, polystyrene is more (by approximately 3 ×) resistant to dry etching than PMMA. Its major drawback is its low sensitivity compared with PMMA, which would limit its application to small scale nano-patterning.

## 2. Experiment

Polystyrene powder with a molecular weight of 2000 g/mol (Mw/Mn = 1.10) was purchased from Alfa Aesa, and dissolved in chlorobenzene with a concentration of 1.2 w/v%, which gave a film thickness of 30 nm, as measured by atomic force microscope (AFM), after spin-coating at 2000 rpm for 40 s. The silicon wafer was cleaned using acetone and 2-proponol, followed by short exposure to oxygen plasma. After spin coating, the film was baked at 60°C for 1 h on a hotplate. Unlike the high molecular weight polystyrene, the low molecular weight polystyrene film was found to be unstable, forming a non-uniform "broken" film when baked at higher temperatures (e.g., 80°C). In addition, its adhesion to the silicon substrate was not as strong as PMMA. Therefore, in order to obtain reproducible uniform film, we coated a thin layer antireflection coating (ARC, from Brewer Science), which was further thinned to < 15 nm by oxygen reactive ion etching with 20 W power and 20 mTorr pressure. This crosslinked and insoluble thin under-layer would not affect the pattern transfer by liftoff; although due to lateral etch, certain critical dimension loss is expected when transferring the pattern by direct etch. Other adhesion promoters, such as a self-assembled monolayer or thin/thinned PMMA film, might also improve the adhesion of polystyrene to the silicon substrate.

Exposure was performed using a LEO 1530 field emission SEM equipped with a Nabity nanometer pattern generation system at acceleration voltages of 20 and 5 kV. The beam currents were about 20 pA at 20 kV and 10 pA at 5 kV. For high-resolution study, the lines were exposed as single-pass lines with beam step size 3 nm, and dots as zero-dimensional dots. After exposure, the samples were developed using various solvent developers for 90 s at room temperature or 50°C, followed by a 2-propanol rinse. As crosslinked polystyrene is insoluble, in principle, all solvents that can dissolve (un-exposed) polystyrene can be used as developer. In this study, we have developed the samples using xylene (o-, m-, p-mixed), chlorobenzene, and cyclohexane.

## 3. Results and discussion

**Figure 1:**
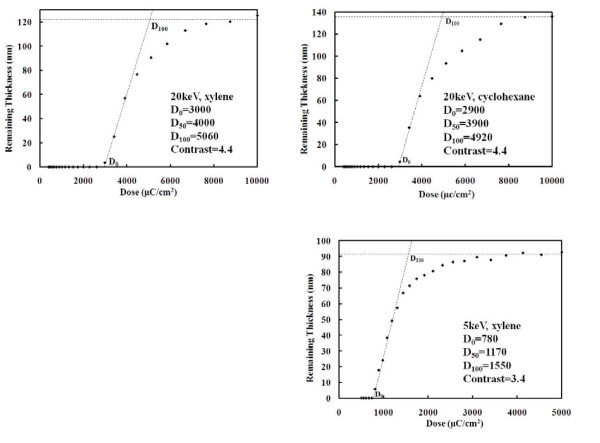
**Contrast curves for polystyrene exposed at 20 and 5 keV, and developed by xylene and cyclohexane for 90 s at room temperature**.

Figure 1 shows the contrast curves for 2000 g/mol polystyrene resist exposed at 20 and 5 keV, using a relatively thick film (125, 135, and 92 nm), which gave more accurate measurement by AFM. Here, in the contrast curves, *D*_0 _and *D*_100 _are the intersections of the line having the highest slope with the zero and full resist thickness lines, respectively. The contrast for exposure at 20 keV, defined as γ = [log(*D*_100_/*D*_0_)]^-1^, is calculated to be 4.4 for both xylene and cyclohexane developers, which is higher than the contrast for ZEP520 resist developed at room temperature [18]. However, the sensitivity for polystyrene resist is rather low with *D*_50 _≈ 4000 μC/cm^2^, which would limit its application to small scale nano-patterning in R&D. The threshold dose where the contrast curve starts to rise (*D*_0_) is the "gel point" that is roughly inversely proportional to the molecular weight for simple negative polymer resists according to the Charlesby theory [19]. This is because the number of crosslinks necessary to make the resist insoluble in the developers decreases with higher molecular weight. We also developed the resist using chlorobenzene but found no apparent difference (the contrast curve is not shown). As for the development temperature, it is well known that generally cold development improves the positive resist contrast and resolution [18,20], whereas hot development increases the contrast for negative resists like HSQ [21]. However, we found no evident improvement for polystyrene (negative) resist development at an elevated temperature of 50°C. One way to alleviate the issue of low resist sensitivity is to carry out exposure at low beam energy such as 5 keV, and the sensitivity was indeed increased to *D*_50 _= 1170 μC/cm^2^. This is in fair agreement with the fact that sensitivity is roughly inversely proportional to the beam energy (*E*) as predicted by the Bethe equation for electron energy loss (*E*_loss_) in the resist: *E*_loss _∞ 1/*E *log(α*E*) with α being a constant. Sensitivity can be further increased using higher molecular weight polystyrene, but at a cost of reduced resolution. When exposed at 5 keV, the contrast is reduced to 3.4, which is close to the ZEP520 resist developed at room temperature [18]. The sensitivity and contrast for 5 keV exposure is expected to be similar for cyclohexane and chlorobenzene developers, as it is for the case of 20 keV exposure. As seen below and pointed out also by Cord et al. [13], the reduced contrast did not seriously affect the resist resolution.

**Figure 2:**
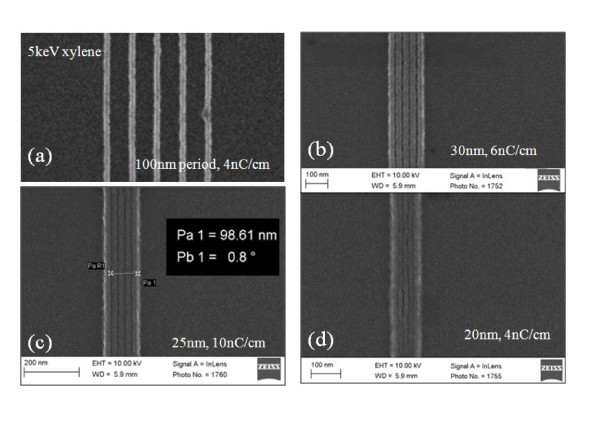
**Dense line array with a period of (a) 100 nm; (b) 30 nm; (c) 25 nm; and (d) 20 nm**. The polystyrene resist was exposed at 5 keV and developed using xylene for 1.5 min at room temperature. The pattern heights measured by AFM are in the range of 25-28 nm that is close to the original film thickness.

**Figure 3:**
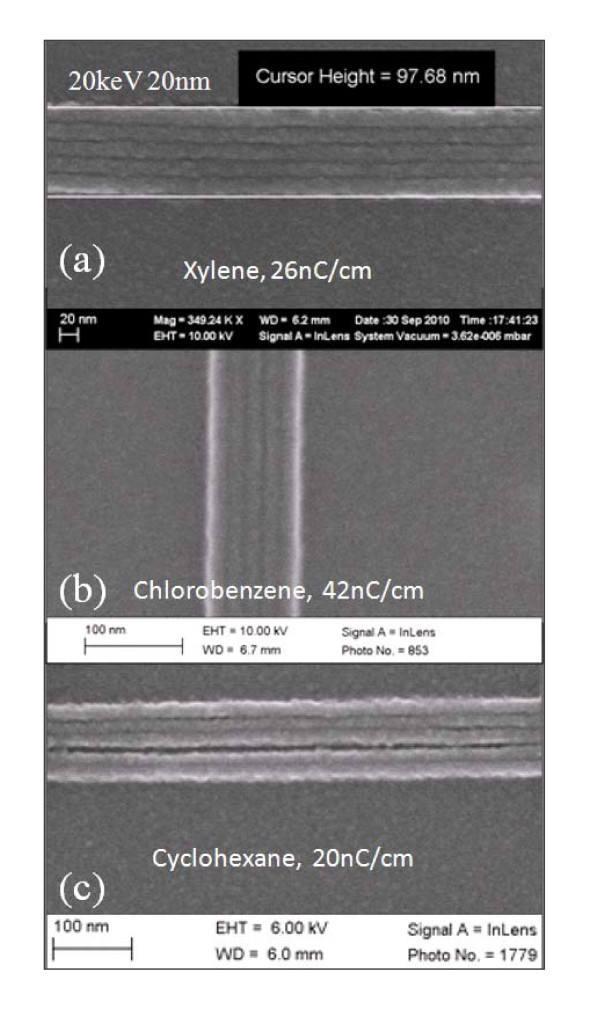
**Dense line arrays with a period of 20 nm exposed at 20 keV and developed at room temperature for 90 s using (a) xylene; (b) chlorobenzene; and (c) cyclohexane**. The lines in (c) collapsed due to capillary force during resist drying.

To study the ultimate resolution (half-pitch) of this resist, we exposed dense line arrays and dot arrays using 30-nm-thick polystyrene at 20 and 5 keV. Thin resist is generally used for high resolution patterning in to reduce the effect of capillary force during resist drying, which leads to pattern collapse (unless using critical point drying [12]), and the forward scattering of electrons that is more serious for thicker resist [13]. Note that even thinner resist was used for most previous high resolution studies on HSQ and calixarene resists. For 30-nm polystyrene, the forward scattering range is estimated to be 5 and 8 nm at 20 and 5 keV, respectively [13], which are both very low (yet slightly larger than or comparable to the beam spot size). Therefore, it is expected that EBL at 5 keV can achieve the same resolution as 20 keV, but with the additional benefit of considerably increased resist sensitivity. Figure 2 shows line array patterns of 100, 30, 25, and 20-nm periods developed by xylene for 90 s at room temperature. Line doses ranging from 4 to 10 nC/cm all resulted in well-defined patterns. The dose window is expected to be much narrower when exposing large area (> 1 μm × 1 μm) line array due to significant exposure from backscattered electrons. The next period in the experiment, 15 nm, was not well defined. The effort toward dense line array patterning by EBL has been driven by the fabrication of X-ray zone plates where the X-ray imaging resolution is close to the half-pitch of the outmost zones. Previously, the densest line array pattern demonstrated using organic resist was 24-nm period using ZEP resist developed at low temperatures [20] (as mentioned above, the record for inorganic HSQ resist is 9-nm period). As expected and shown in Figure 3, for exposure at 20 keV, a similar high resolution of 20-nm period could be achieved when using all the three developers (xylene, chlorobenzene, and cyclohexane) that are studied.

**Figure 4:**
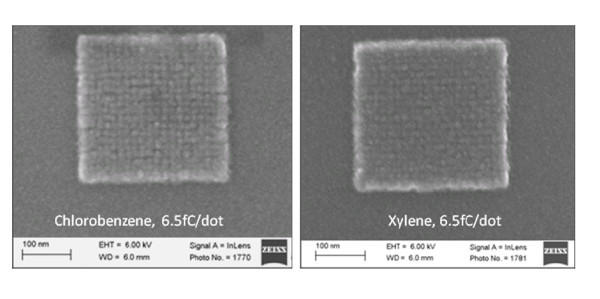
**Dense 2D dot array with a period of 15-nm exposure at 5 keV and developed by chlorobenzene and xylene for 1.5 min at room temperature**.

For dot array patterns, the densest array for which the dot is still fairly well defined is with a 15-nm period (Figure 4), which is believed to be the highest pattern density ever obtained using organic EBL resists. Here, the array was exposed at 5 keV and developed by xylene and chlorobenzene for 90 s at room temperature. The effort toward dense 2D array pattern has been driven by the fabrication of bit-patterned media [22], and the previously evaluated array periods of 18 nm (corresponding to 2.0 Tbits/in^2^) using organic ZEP resist, and 12 nm using inorganic HSQ resist have been achieved [23].

## 4. Conclusions

We studied the exposure behavior of the negative EBL resist polystyrene. It demonstrated fairly well-defined patterns of 20-nm-period line arrays and 15-nm-period dot arrays, which are the densest patterns ever achieved using organic EBL resists. Such dense patterns can be achieved both at 20 and 5 keV beam energies, using all the three developers that were studied. The contrast for polystyrene is comparable to that of other popular resists like ZEP and PMMA, but its sensitivity is low. In addition to its high-resolution capability, polystyrene is a simple and low-cost resist with easy process control and practically unlimited shelf life. It is also considerably more resistant to dry etching than PMMA. It would find applications where negative resist is prefered and exposure time is not a major concern.

## Abbreviations

AFM: atomic force microscope; ARC: antireflection coating; EBL: electron beam lithography; FIB: focused ion beam; HSQ: hydrogen silsesqioaxene; NIL: nanoimprint lithography; PMMA: poly(methyl methacrylate).

## Competing interests

The authors declare that they have no competing interests.

## Authors' contributions

SM and CC carried out the experiment. BC and MY designed the study. BC analyzed the data and prepared the manuscript.
